# Genetic Analysis in a Familial Case With High Bone Mineral Density Suggests Additive Effects at Two Loci

**DOI:** 10.1002/jbm4.10602

**Published:** 2022-02-18

**Authors:** Núria Martínez‐Gil, Diana Ovejero, Natalia Garcia‐Giralt, Carlos David Bruque, Leonardo Mellibovsky, Xavier Nogués, Raquel Rabionet, Daniel Grinberg, Susanna Balcells

**Affiliations:** ^1^ Department of Genetics, Microbiology and Statistics, Faculty of Biology Universitat de Barcelona, Centro de Investigación Biomédica en Red de Enfermedades Raras (CIBERER), instituto de investigación biomédica básica de la Universidad de Barcelona (IBUB), Institut de Recerca Sant Joan de Déu (IRSJD) Barcelona Spain; ^2^ Musculoskeletal Research Group IMIM (Hospital del Mar Medical Research Institute), Centro de Investigación Biomédica en Red en Fragilidad y Envejecimiento Saludable (CIBERFES), Instituto de Salud Carlos III (ISCIII) Barcelona Spain; ^3^ Unidad de Conocimiento Traslacional Hospitalaria Patagónica Hospital de Alta Complejidad Servicio de Atención Médica Integral para la Comunidad (SAMIC) ‐ El Calafate El Calafate Argentina

**Keywords:** DISEASES AND DISORDERS OF/RELATED TO BONE, GENETIC RESEARCH, MOLECULAR PATHWAYS‐REMODELING

## Abstract

Osteoporosis is the most common bone disease, characterized by a low bone mineral density (BMD) and increased risk of fracture. At the other end of the BMD spectrum, some individuals present strong, fracture‐resistant, bones. Both osteoporosis and high BMD are heritable and their genetic architecture encompasses polygenic inheritance of common variants and some cases of monogenic highly penetrant variants in causal genes. We have investigated the genetics of high BMD in a family segregating this trait in an apparently Mendelian dominant pattern. We searched for rare causal variants by whole‐exome sequencing in three affected and three nonaffected family members. Using this approach, we have identified 38 rare coding variants present in the proband and absent in the three individuals with normal BMD. Although we have found four variants shared by the three affected members of the family, we have not been able to relate any of these to the high‐BMD phenotype. In contrast, we have identified missense variants in two genes, *VAV3* and *ADGRE5*, each shared by two of out of three affected members, whose loss of function fits with the phenotype of the family. In particular, the proband, a woman displaying the highest BMD (sum *Z*‐score = 7), carries both variants, whereas the other two affected members carry one each. *VAV3* encodes a guanine‐nucleotide‐exchange factor with an important role in osteoclast activation and function. Although no previous cases of *VAV3* mutations have been reported in humans, *Vav3* knockout (KO) mice display dense bones, similarly to the high‐BMD phenotype present in our family. The *ADGRE5* gene encodes an adhesion G protein‐coupled receptor expressed in osteoclasts whose KO mouse displays increased trabecular bone volume. Combined, these mouse and human data highlight *VAV3* and *ADGRE5* as novel putative high‐BMD genes with additive effects, and potential therapeutic targets for osteoporosis. © 2022 The Authors. *JBMR Plus* published by Wiley Periodicals LLC on behalf of American Society for Bone and Mineral Research.

## Introduction

Osteoporosis, the most common bone disease, is characterized by a reduced bone mineral density (BMD) and increased risk of fracture. Osteoporotic fractures and their treatments are accompanied by high morbidity/mortality and a high sociosanitary cost, which increases with increasing life expectancy. In recent times, treatments have been designed based on targets derived from the genetic study of rare monogenic diseases.^(^ ^1^, ^2^, ^3^, ^4^, ^5^ ^)^ In particular, some diseases characterized by an elevated BMD are of special interest to obtain novel therapeutic targets to improve BMD and skeletal architecture. The high BMD phenotypes can be categorized according to the underlying biological mechanism: (i) decreased bone resorption; (ii) enhanced bone formation; and (iii) alteration of bone turnover rate.^(^ ^6^ ^)^ It is worth noting that a high BMD is not always associated with a lower risk of fractures. For example, in osteopetrosis, a category of high‐BMD disease due to decreased bone resorption, bone is brittle and fractures easily.^(^ ^7^ ^)^ Some of the genes identified as causing osteopetrosis belong either to the nuclear factor κB (NFκB) signaling pathway, which is essential for the differentiation of osteoclasts, or to the pathway for acidification of the extracellular compartment.^(^ ^7^ ^)^ On the other hand, sclerosteosis, van Buchem disease and the high bone mass (HBM) phenotype are caused by enhanced bone formation, and are characterized by unusually dense bones and a very strong skeleton, resulting in a dramatic decrease in fracture risk.^(^ ^6^, ^8^ ^)^ Sclerosteosis and van Buchem disease are two rare autosomal recessive diseases caused by mutations in the genes for the Wnt pathway inhibitor *SOST* or for its coreceptor *LRP4*.^(^ ^9^, ^10^, ^11^, ^12^, ^13^, ^14^ ^)^ These patients present hyperostosis of the whole skeleton, but most prominently at the skull, mandible, and long bones, often leading to symptomatic manifestations including hearing loss, facial palsy, and severe headache, as a result of nerve compression. In contrast, HBM patients present milder manifestations such as enlarged mandible and torus palatinus, and in most cases, they either do not require treatment, or are asymptomatic. Thus, the HBM phenotype is normally detected casually through BMD measured by bone densitometry (dual‐energy X‐ray absorptiometry [DXA]). However, there is no consensus about the DXA cutoff value for a HBM diagnosis. Although some authors use *Z*‐score ≥+2.5 at either lumbar spine (LS) or femoral neck (FN),^(^ ^15^ ^)^ others consider obtaining a value greater than four when performing the sum of *Z*‐scores measured at the LS and FN.^(^ ^16^ ^)^ Gain of function mutations in *LRP5* and *LRP6* have been described to cause HBM.^(^ ^16^, ^17^, ^18^, ^19^ ^)^ These variants produce a loss of affinity for the extracellular inhibitors Dickkopf‐1 (DKK1) and *SOST*, thus preventing internalization of low‐density lipoprotein receptor‐related protein 5/6 (LRP5/6).^(^ ^16^, ^18^, ^19^, ^20^, ^21^, ^22^, ^23^, ^24^ ^)^ All the *LRP5/6*‐HBM mutations described are located in the first β‐propeller domain, which interacts with these extracellular Wnt pathway inhibitors.^(^ ^25^, ^26^, ^27^ ^)^ In this regard, we have recently described two rare missense mutations in *DKK1* (p.Tyr74Phe and p.Arg120Leu) in two HBM women,^(^ ^28^, ^29^ ^)^ and validated them functionally by demonstrating their partial loss of function.^(^ ^30^ ^)^ In addition to the mutations in canonical Wnt pathway genes, mutations in other genes have been found as potential candidates to cause the HBM phenotype. This is the case of a rare dominant missense mutation in *SMAD9* (p.Leu22Pro), which was found cosegregating with this trait in a family and in two unrelated patients.^(^ ^31^ ^)^ SMAD9 acts by inhibiting BMP‐dependent target gene transcription in osteoblasts, thus limiting osteoblast activity.^(^ ^32^ ^)^ In addition to these monogenic forms, the HBM phenotype can also be polygenic. A recent study of a HBM cohort has found an enrichment for common protective variants at BMD genomewide association study (GWAS) loci as compared with a reference cohort of normal BMD.^(^ ^33^ ^)^ Although a proportion of all HBM cases are expected to be monogenic, in most of these cases causative mutations are yet to be identified. Likewise, the implication of a combination of a small number of highly penetrant variants generating digenic, trigenic, or oligogenic patterns of inheritance, modified by common variants with small effects has not been investigated, yet.

Here, we have undertaken the genetic analysis of rare coding variants in a family segregating a high‐BMD phenotype in an apparently Mendelian fashion and for which we had already collected evidence against polygenic inheritance.^(^ ^28^ ^)^

## Subjects and Methods

### Ethics statement

Both the Bioethics Committee of Universitat de Barcelona and the Clinical Research Ethics Committee of Parc de Salud Mar have emitted favorable bioethical statements regarding the present research. Written informed consents were obtained from the participants in both instances. Blood samples and written informed consent were obtained in accordance with the regulations of the Clinical Research Ethics Committee of Parc de Salut Mar.

### Biological samples

**Fig. 1:**
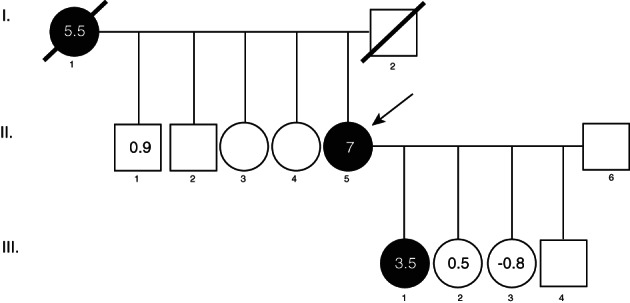
Pedigree of the family segregating a high BMD phenotype. Black filled symbols denote individuals presenting with the trait. The numbers inside the symbols correspond to the sum BMD *Z*‐scores (LS+FN).

The study includes six family members, three with high BMD and three with normal BMD (Fig. 1). For all the participating family members, clinical history, a blood test, including a complete biochemical analysis of relevant parameters (complete blood count [CBC], kidney function, liver biology, thyroid function) and BMD quantification at the lumbar spine (L_1_–L_4_ [LS]) and femoral neck (FN), performed by dual‐energy X‐ray absorptiometry scans (DXA; QDR 4500 SL; Hologic, Inc., Marlborough, MA, USA) were available. All DXA measurements were performed prior to any treatment that could affect bone mass. In addition, mineral metabolism parameters including calcium, phosphate, vitamin D, and bone remodeling markers, including serum C‐terminal telopeptide of type I collagen (CTX) as a bone resorption marker, bone specific alkaline phosphatase (BSAP), and N‐terminal propeptide of type I procollagen (P1NP) as bone formation markers, were measured in the proband and her three daughters (II.5 and III.1, III.2, and III.3), at the reference laboratory of the Hospital del Mar (Barcelona). Genomic DNA from all the family participants was extracted from peripheral blood leukocytes using the Wizard® Genomic DNA Purification Kit (Promega, San Luis Obispo, CA, USA), according to the manufacturer's instructions.

### Whole‐exome sequencing and filtering

**Fig. 2:**
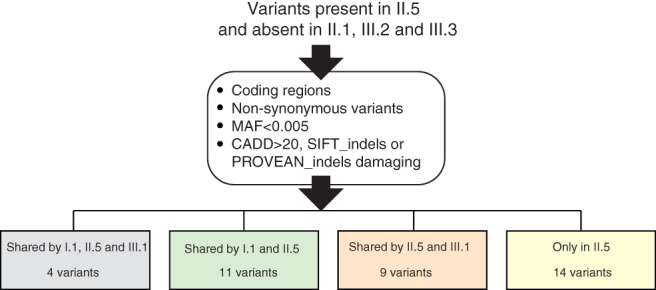
Variants found in the proband and absent in the three unaffected individuals of the family. Pipeline filtering scheme. Variants shared among the three members with high BMD (gray), between the proband and her mother (green), between the proband and her daughter high BMD (orange), and only in the proband (yellow). Details of these variants are found in Supplementary Tables S2–S5, respectively.

Whole‐exome sequencing (WES) of I.1, II.1, II.5, III.1, III.2, and III.3 (Fig. 1 and Table 1) was performed at Centre Nacional d'Anàlisi Genòmica (CNAG, Barcelona, Spain). Shortly, DNA was enzymatically fragmented and libraries were constructed and hybridized against the Nimblegene Human Clinical Exome Capture + Mitochondrial DNA (I.1, II.1, II.5) or KAPA HyperExome + mitochondrial DNA (III.1, III.2, and III.3) probesets. Captured fragments were sequenced in an Illumina NovaSeq 6000 sequencer (Illumina, San Diego, CA, USA). Quality control stats from the six WES analyses are detailed in Supplementary Table S1. The reads were then aligned to the hg38 reference genome with burrows‐wheeler aligner‐mem, duplicate‐marked, recalibrated, and sorted before calling variants with Genome Analysis Toolkit (GATK, Cambridge, MA, USA) haplotype caller (V4) following GATK standard parameters. After quality‐filtering following GATK recommended hard filters (https://gatk.broadinstitute.org/hc/en-us/articles/360035890471-Hard-filtering-germline-short-variants), variants were annotated with the Variant Annotation and Filter Tool (Varaft, Aix Marseille University, UMR 1251, France; https://varaft.eu/),^(^ ^34^ ^)^ and prioritized under the hypothesis of an autosomal dominant segregation. We therefore filtered by variants present in proband II.5 and absent in II.1, III.2, and III.3 (Fig. 2 and Supplementary Fig. S1). Variants located outside of the coding region (intergenic, 5′ and 3′ untranslated region [UTR], upstream, downstream, noncoding RNA [ncRNA], or unknown variants), intronic variants not predicted to affect the splice site, synonymous variants, those with a minor allele frequency >0.005, those with a combined annotation‐dependent depletion (CADD; http://cadd.gs.washington.edu) pathogenicity scores <20 for single‐nucleotide variant (SNV) or the sorting intolerant from tolerant (SIFT) indel (Bioinformatics Institute, Singapore; https://sift.bii.a-star.edu.sg/) or Protein Variation Effect Analyzer (PROVEAN) indel pathogenicity score neutral (J. Craig Venter Institute, La Jolla, CA, USA; http://provean.jcvi.org/) for indels, and those in genes enriched in missense variants according to the Genome Aggregation Database (gnomAD V2.1.1; Broad Institute, Cambridge, MA, USA) were filtered out (Fig. 2 and Supplementary Fig. S1). Of the resulting 38 variants, we have determined 4 which were present in the three women with high BMD (I.1, II.5, and III.1), 11 in I.1 and II.5 only, 9 in II.5 and III.1 only, and 14 which were only present in proband II.5 (Fig. 2 and Supplementary Tables S2–S5). Then, these 38 variants were prioritized based on gene functionality information (including known gene function, phenotype of animal models, human diseases, and association in BMD GWAS), obtained from public databases (Musculoskeletal Knowledge Portal, Uniprot, OMIM, and Pubmed) resulting in a list of six variants in six different genes (Table 2). We have verified the presence and absence of these six variants in all the participant members by Sanger sequencing at the CCiTUB genomics service (Genòmica, Parc Cientific, Barcelona, Spain) using the BigDye Terminator v3.1 Cycle Sequencing Kit, followed by detection on automated capillary sequencer models 3730 Genetic Analyzer and 3730xl Genetic Analyzer (all from Thermo Fisher Scientific, Waltham, MA, USA).

### Protein structure analysis

Molecular homology modeling (MHM) was performed for VAV3 protein (UniProt_IDQ9UKW4) between amino acid residues 1–560 and the predicted structure for ADGRE5 (aka CD97) protein (UniProt_ID P48960) between amino acid residues 492 and 835. The evaluation criteria to select the template for the MHM was: (i) protein sequence identity as template 55%; (ii) existence of X‐ray crystal; (iii) source organism “*Homo sapiens*”; and (iv) chain length and amount of residues of each template with respect to sequence identity and gaps. The alignment between templates and target sequences was performed with the Structural alignments (Expresso extension) in the T‐Coffee web server (Center for Genomic Regulation, Barcelona, Spain; https://www.tcoffee.org/Projects/tcoffee/index.html)^(^ ^35^, ^36^ ^)^ and MEGA X software (Molecular Evolutionary Genetics Analysis; https://www.megasoftware.net/home), taking into account the secondary structures and topology of the regions.^(^ ^37^ ^)^ The MHM was generated using MODELLER (Departments of Biopharmaceutical Sciences and Pharmaceutical Chemistry, and California Institute for Quantitative Biomedical Research, University of California San Francisco, San Francisco, CA, USA; https://salilab.org/modeller/).^(^ ^38^ ^)^ The templates for the VAV3 MHM were 3KY9; 2VRW; 6NF1; 6NFA; 6NEW; and 3BJI. The models were first optimized with the variable target function method with conjugate gradients, and then refined using molecular dynamics with simulated annealing.^(^ ^39^ ^)^ Model quality evaluation was performed using Discrete Optimized Protein Energy (DOPE) = −79487.^(^ ^40^ ^)^ The model is available in ModelArchive at https://modelarchive.org/doi/10.5452/ma‐ak41q. The structure analysis of ADGRE5 (CD97) was obtained from AlphaFold2 protein structure database prediction (European Molecular Biology Laboratory–European Bioinformatics Institute [EMBL‐EBI], Hinxton, UK; https://www.alphafold.ebi.ac.uk/).^(^ ^41^ ^)^ The University of California, San Francisco (UCSF) Chimera program^(^ ^42^ ^)^ and the back‐bone dependent rotamer library were used for structural interpretation and visualization.^(^ ^43^ ^)^ The line protein graphs in figures were generated with the PyGame library in the Python 2.7 programming language (Python Software Foundation, Troisdorf, Germany; http://www.python.org).^(^ ^44^ ^)^

## Results

### Case report

The proband (II.5 in Fig. 1) was first seen in our Mineral Metabolism clinic for osteoporosis assessment due to postmenopausal status at age 66 years. She was then invited to enroll in the cohort of Spanish postmenopausal women (BARCOS) from the Barcelona area.^(^ ^45^, ^46^ ^)^ Within BARCOS, a total of 1600 lumbar spine (LS) and femoral neck (FN) BMD measurements were analyzed in order to identify those women with extreme BMD values. The proband presented a sum of *Z*‐score = 7 (*Z*_LS_ = 4.6 and *Z*_FN_ = 2.4; Table 1). Analysis of the BMD‐risk alleles (performed in a previous study^(^ ^28^ ^)^), in which the proband is included as patient HBM9, yielded a surprisingly high number of variants associated to low BMD, which would predict a low bone mass phenotype due to the incremental effects of each of these risk alleles. Because of this apparent contradictory result, we hypothesized that the proband might be carrying additional unknown rare and penetrant variants, which would be responsible for her high‐BMD phenotype and might segregate in a Mendelian fashion in her family. Subsequently, we analyzed her relatives, including her mother (I.1), brother (II.1), and three daughters (III.1, III.2, and III.3). DXA analysis revealed that the mother, 84 years old at the time of DXA, presented a sum *Z*‐score of 5.5 (*Z*_LS_ = 3.3 and *Z*_FN_ = 2.2; Table 1) thus, revealing a high BMD (Fig. 1). Noteworthy, her L_3_ was excluded from this analysis given the presence of grade I wedge compression fracture in a lateral X‐ray performed at that time. The abdominal aorta also appeared calcified, but according to the literature, we did not consider that this finding should significantly increment BMD.^(^ ^47^ ^)^ In addition, osteophytes and other osteoarthritis (OA)‐related signs were practically absent. Also, following the International Society for Clinical Densitometry DXA quality control position statement,^(^ ^48^ ^)^ there was not more than one standard deviation (SD) difference in individual *T*‐scores between adjacent lumbar vertebrae (and between L_4_ and L_2_, given that L_3_ had been excluded). Taking all of this into account, we considered that artifacts did not influence significantly this patient's BMD. One of the proband's daughters (III.1), who was 33 years old at the time she underwent DXA, presented a sum *Z*‐score of 3.5 (*Z*_LS_ = 1.3 and *Z*_FN_ = 2.2; Table 1). This value is lower than the proband and her mother, but considering the variability in the threshold to define high BMD and the fact that two members in the family have high BMD, we considered daughter III.1 as a high‐BMD patient. The other two daughters, III.2 and III.3, presented sum *Z*‐scores of 0.5 (*Z*_LS_ = −0.2 and *Z*_FN_ = 0.7; Table 1) and −0.8 (*Z*_LS_ = −0.4 and *Z*_FN_ −0.4; Table 1), respectively, and the proband's brother, II.1, presented a sum *Z*‐score of 0.9 (*Z*_LS_ = −0.1 and *Z*_FN_ = 1; Table 1), all rated as normal. Besides the aforementioned issues in the mother of the proband (I.1), presence of potential artifacts at the lumbar spine and femoral neck that could overestimate BMD were ruled out in all the remaining cases. In addition, the patients did not exhibit any evident feature linked to osteopetrosis and/or sclerosteosis, and we also excluded potential confounders that can result in elevated BMD such as Paget disease, acromegaly, or hepatitis C virus (HCV) infection. Finally, all biochemical markers of bone turnover fell within the normal range, although the CTX marker for II.5 and III.1 was in the lower end of the reference range (Table 1).

**Table 1:** BMD *Z*‐score Bone Turnover Markers and Vitamin D Levels of All the Family Participants

Participant	*Z*‐score BMD		Bone turnover markers and vitamin D			
	LS	FN	P1NP (16–74 ng/mL)	BSAP (4.3–20.1 μg/L)	CTX (0.01–1.008 ng/mL)	Vitamin D (30–150 ng/mL)
I.1	3.3	2.2	NA	NA	NA	NA
II.1	−0.1	1.0	NA	8.65	NA	NA
II.5	4.6	2.4	27.5	11.8	0.085	NA
III.1	1.3	2.2	24.4	9.14	0.110	26
III.2	−0.2	0.7	32.7	10.40	0.269	35
III.3	−0.4	−0.4	55.9	8.50	0.248	44

BSAP = bone‐specific alkaline phosphatase; CTX = C‐terminal telopeptides of type I collagen; FN = femoral neck; LS = lumbar spine; P1NP = type I serum procollagen, N‐terminal propeptide of type I procollagen.

We performed WES analysis for the three high BMD samples (proband II.5, her mother I.1, and her daughter III.1), the proband's brother (II.1), and the two daughters (III.2 and III.3) whose BMD values were in the normal range. Our objective was to identify a variant common to the three high‐BMD women. We first selected variants present in the proband II.5, whose BMD is the highest in the family, and absent in the three unaffected members (II.1, III.2, and III.3) (Fig. 2). After the applied filters (Fig. 2 and Supplementary Fig. S1), we identified 38 variants present in proband II.5. Four of these were common to the three high‐BMD women (*ARMC9*, *RPUSD1*, *TBL3*, and *HSPA12B*; Supplementary Table S2). However, we were not able to retrieve convincing evidence in the literature or in databases to suggest the involvement of any of these four genes with the elevated BMD phenotype of the family. We then inspected the remaining 34 variants, of which 11 were common to the proband and her mother (II.5 and I.1; Supplementary Table S3), 9 were shared by the proband and her daughter with high BMD (II.5 and III.1; Supplementary Table S4) and 14 were only present in the proband (Supplementary Table S5).

Two variants in two genes, *VAV3* and *ADGRE5* (Table 2), caught our attention by their known function in bone biology and the bone phenotypes of existing knockout (KO) animal models (see Discussion). The *VAV3* variant is shared by the proband and her mother, whereas that of *ADGRE5* is shared by the proband and her daughter.

**Table 2:** Candidate Variants To Be Responsible of the High BMD in the Family

Gene	Variant	rs number	Cosegregation	BMD GWAS	Disease	Pathogenicity prediction				
						CADD	PP	PV	SIFT	MAF gnomAD
*AMOTL1*	p.Y605S^a^	NA	I.1 and II.5	N	N	26.4	D	D	D	NA
*VAV3*	p.T124I	rs200980013	I.1 and II.5	N	N	23.1	B	N	T	0.00015
*CDK5RAP3*	p.D271N^b^	rs140552517	I.1 and II.5	Y	N	22.8	B	D	T	0.00238
*ADGRE5*	p.R794W^c^	rs369617596	II.5 and III.1	N	N	28	D	D	D	0.00013
*GLI1*	p.R510W^d^	rs149817893	II.5 and III.1	N	PA	24.4	B	N	D	0.00268
*PLXNB2*	p.E1804K	rs149124212	II.5	Y	N	28.1	D	N	D	0.00401

BMD GWAS = genes associated with BMD in GWAS (N = no; Y = yes); CADD = http://cadd.gs.washington.edu; Cosegregation = family members in whom the variant is present; Disease = gene associated with human diseases in OMIM (N = no; PA = polydactyly); MAF = minor allele frequency from gnomAD V2.1.1; PP = Polyphen‐2 (http://genetics.bwh.harvard.edu/pph2/; B = benign; D = probably damaging); PV = PROVEAN (http://provean.jcvi.org/; D = deleterious; N = neutral); SIFT = sorting intolerant from tolerant (https://sift.bii.a‐star.edu.sg/; D = deleterious; T = tolerated).

^a^ NM_130847.3.

^b^ NM 176096.3.

^c^ NM_078481.4.

^d^ NM_005269.3.

**Fig. 3:**
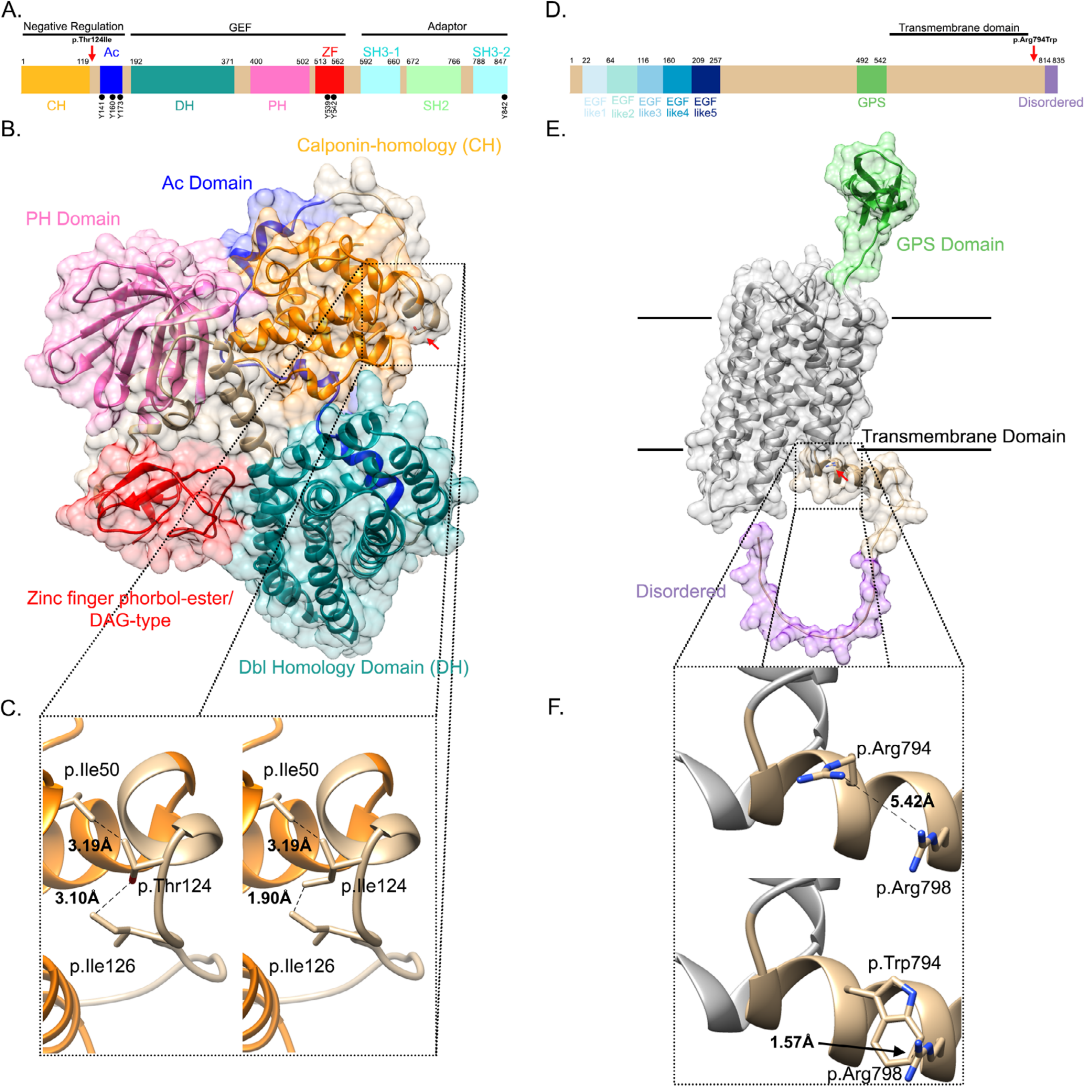
Domain architecture, mapping, and structural analysis of variants in VAV3 and ADGRE5 (CD97). (*A*) Linear representation of the VAV3 protein with its domains and regions (from UniProt): Calponin‐homology (CH, orange), Acidic domain (Ac, dark blue), Dbl homology domain (DH, dark cyan), Pleckstrin homology domain (PH, pink), Zing finger phorbol‐ester/ DAG‐type (red), SH3‐1 nad SH3‐2 domains (cyan), SH2 domain (light green). Relevant tyrosine residues (Y) are depicted below the scheme with black dots. Three main functions of VAV3 (negative regulation, guanine exchange factor and adaptor) are ascribed to particular groups of domains, as indicated. The red arrow signals the position of the p.Thr124Ile variant found in two patients from this family. (*B*) Human VAV3 molecular homology model, with the representation of domains. (*C*) In zoom we show the p.Thr124Ile variant analysis. (*D*) Linear representation of the C97 Protein with its domains (from UniProt): EGF like 1 to EGF like 5 (gradient of blues), GPS Domain (Green), Disordered tail (violet), and Transmembrane Domain (black line). The red arrow indicates the position of the p.Arg794Trp variant shared by two patients in the family. (*E*) Human CD95 protein structure prediction with AlphaFold2, with the representation of domains. (*F*) In zoom we show the p.Arg794Trp variant analysis.

To gain additional insight into the nature of these two variants, we performed molecular homology modeling for the VAV3 protein and a predictive structure for the ADGRE5 (CD97) protein. The variant p.Thr124Ile in VAV3 lies next to the calponin homology (CH) domain (Fig. 3*A*) between the 9th and 10th α‐helices, altering the folding of this loop and compromising the stability of the region (Fig. 3*B*). The variant affects the region with a steric effect between the Ile50 in the CH domain and Ile126. In particular we observe a reduction (of 1.2 Å) in the distance to residue Ile126 for the Ile124 variant, compared to the wild‐type Threonine 124 (Fig. 3*C*). Furthermore, the stability for this variant, as calculated with FoldX, was ΔΔG = 1.14 ± 0.03 kcal/mol (close to the 1.6 threshold), and the main component of instability were van der Waals forces, reinforcing the idea of a putative steric effect. The ADGRE5 (CD97) variant p.Arg794Trp lies between the transmembrane domain and the disordered region (Fig. 3*D*) in the C‐terminal amphipathic α‐helix of the protein on the cytosolic side (Fig. 3*E*). The replacement of Arginine 794 by Tryptophan has a steric effect between residues 794 and 798. We observe an important reduction (of 3.85 Å) in the distance between these residues (Fig. 3*F*). However, the stability for the ADGRE5 (CD97) mutation, as calculated with FoldX, was within the normal range (ΔΔG = 0.372 ± 0.32 Kcal/mol).

In addition to *VAV3* and *ADGRE5*, variants in four other genes (*AMOTL1*, *CDK5RAP3*, *GL1*, and *PLXNB2*; Table 2) could also play a role in the phenotype (see below in the discussion section). Yet, we hypothesize that the two rare variants in *VAV3* and *ADGRE5* are the most likely candidates to be involved in the inherited high‐BMD phenotype in this family.

## Discussion

Due to the extremely high frequency of osteoporosis in the general population, which increases with life expectancy, it is vital to find appropriate treatments. In this context, it is of special interest to study the genetic basis of unique high‐BMD phenotypes. In particular, a gene whose loss‐of‐function causes a high‐BMD phenotype without any other secondary complication represents an ideal candidate for a therapeutic target.

Here we report a family with an unexplained high BMD, apparently inherited in an autosomal dominant manner, and whose proband was shown to carry an excess number of osteoporosis risk alleles.^(^ ^28^ ^)^ The family includes a proband, her mother, and her daughter displaying high BMD, and a brother and two daughters of the proband displaying normal BMD values. We performed exome sequencing, expecting to find a common variant among the three affected members and absent in the three members with normal BMD, which would explain the phenotype. We found four such variants, in *ARMC9*, *RPUSD1*, *TBL3*, and *HSPA12B* (Supplementary Table S2). However, they showed no clear evidence suggestive of a possible causal role in the high‐BMD phenotype. *ARMC9* plays an important role in ciliary stability and function^(^ ^49^ ^)^ and is a cause of Joubert syndrome, a severe neurodevelopment ciliopathy with no reported BMD changes.^(^ ^50^ ^)^ *RPUSD1* has been associated with nevoid basal cell carcinoma syndrome by WES in a Chinese population.^(^ ^51^ ^)^ TBL3 is a nucleolar protein with an important function on cell‐cycle rate during zebrafish development, whose absence affects the size of differentiated tissues but not their specification.^(^ ^52^ ^)^ Finally, HSPA12B is required in angiogenesis to form functional vessels in ischemic tissue.^(^ ^53^ ^)^ In any case, extensive functional studies would be required, including the generation of knockin animals, to fully assess the involvement of any of them.

Then, we considered the possibility that the high‐BMD phenotype could be due to several variants with additive effects in the same direction. For this, we studied the 34 remaining variants present in the proband and absent in the nonaffected relatives (Table 2). Two of them, one shared by the proband and her mother and the other by the proband and her affected daughter, caught our attention for their role in bone metabolism.

The first one is variant p.Thr124Ile in *VAV3*, present in the proband and her mother. VAV3 is a guanine nucleotide exchange factor (GEF) of the Rho family that has an important role in bone resorption. In particular, VAV3 is essential for organizing the osteoclast's cytoskeleton and it has been shown to stimulate osteoclast activation in vitro.^(^ ^54^ ^)^ In addition, the *Vav3*‐KO mouse presents high bone mass, reflecting an impaired osteoclast terminal differentiation and function, and protection against bone loss induced by parathyroid hormone (PTH) or receptor activator of nuclear factor κB ligand (RANKL).^(^ ^54^ ^)^ Furthermore, a GWAS study showed association of *VAV3* with BMD,^(^ ^55^ ^)^ reinforcing its importance in bone metabolism. The VAV3 protein presents a series of domains (Fig. 3*A*–*C*) through which it exerts its two main activities of guanine exchange factor (GEF) and adaptor.^(^ ^56^ ^)^ The catalytic activity of VAV3 is modulated by tyrosine phosphorylation at position Y141. When this tyrosine is not phosphorylated, Dbl homology (DH) and acidic (Ac) domains together with the most N‐terminal CH region contact the catalytic domains, preventing interaction with the substrate and forming an autoinhibitory loop. Interestingly, the mutation identified in the proband maps to the N‐terminal CH region and, presumably, it could disrupt VAV3 activity by permanently forcing its autoinhibition.

The second variant is p.Arg794Trp in *ADGRE5*, present in the proband and her high‐BMD daughter. This gene encodes for CD97, a member of the seven transmembrane epidermal growth factor family of adhesion G protein coupled receptors (GPCRs).^(^ ^57^ ^)^ CD97 has been widely studied for its role in cell adhesion, leukocyte recruitment and migration, and in immune responses due to its high expression at inflammatory sites.^(^ ^58^ ^)^ Along with this role, it has been seen that CD97 acts as a positive regulator of osteoclast differentiation and function thanks to in vitro and in vivo studies, where the KO mouse shows a high BMD attributable to decreased function and number of osteoclasts.^(^ ^59^ ^)^ Three CD97 ligands have been identified, including integrins,^(^ ^60^ ^)^ glycosaminoglycan chondroitin sulfate,^(^ ^61^ ^)^ and CD55.^(^ ^62^ ^)^ Confirming the results found in CD97, the CD55‐KO mouse model also shows a high BMD due to a decrease in osteoclast activity.^(^ ^63^ ^)^ Interestingly, in a recent work from our group exploring a very particular high‐BMD related phenotype, we identified another *ADGRE5* variant, in a nearby residue, also located in the cytoplasmic domain (Ovejero D et al, unpublished data). Although all the pathogenicity predictors used score the variant as damaging, in the structural studies we have carried out with the Alphafold2 program, we have not seen any effect on the stability of the protein. In fact, the variant is found in the eighth alpha helix of the cytoplasmic side of the protein. As has been reviewed,^(^ ^64^ ^)^ this eighth alpha helix of many GPCRs plays important roles in the interaction with other proteins to properly function, including signal transduction. Therefore, it could be hypothesized that this variant may be causing a defect in *ADGRE5* signaling.

Considering this information, it is tempting to speculate that loss of function of VAV3 and/or ADGRE5, due to the p.Thr124Ile and p.Arg794Trp mutations, respectively, could cause a defect in bone resorption leading to a high‐BMD phenotype accompanied by the observed low CTX resorption values. In this same line, the presence of a mild vertebral compression fracture in the mother advocates for a high‐BMD phenotype due to an osteoclastic defect. Fractures secondary to bone brittleness are a classical feature of osteopetrotic syndromes, rather than to elevated BMD values due to supraphysiological bone formation, which is typically associated to decreased fracture risk.^(^ ^65^ ^)^ Additionally, the presence of these two variants in the proband and only one of them in the mother and in the daughter with high BMD could explain the higher BMD found in the proband.

In addition to these two very interesting variants, we would also like to emphasize the presence of other variants in important bone‐related genes, such as *CDK5RAP3*, *AMOTL1*, *GLI1*, and *PLXNB2*, that could play a modifying role in the high‐BMD phenotype of this family.

*LZAP*, aka *CDK5RAP3*, is a tumor suppressor gene, which exerts its function on many signaling pathways such as the inhibition of the NFκB or the activation of the p53 pathways.^(^ ^66^, ^67^ ^)^ In addition, it controls the nuclear localization of β‐catenin through glycogen synthase kinase 3 (GSK3). It could be hypothesized that a missense loss‐of‐function mutation could result in an activation of the Wnt pathway, which in osteoblasts would produce an increase in bone formation, being able to generate a phenotype of high bone mass. *AMOTL1* encodes a protein of the Motin family. This family has an important role in angiogenesis, cell mobility, cell polarity, and cell–cell junctions through different pathways including the canonical Wnt pathway.^(^ ^68^, ^69^ ^)^ Interestingly, Li and colleagues,^(^ ^69^ ^)^ demonstrate that Amotl1 attenuates Wnt/β‐catenin signaling in zebrafish. Thus, a loss of function variant in *AMOTL1* could have a similar effect as proposed for *CDK5RAP3. GLI1* encodes a transcription factor downstream of hedgehog (HH). Interestingly, Indian HH regulates osteoblast differentiation during endochondral bone development in the embryo,^(^ ^70^, ^71^, ^72^ ^)^ and is also involved in bone formation in postnatal mice.^(^ ^73^ ^)^ It could be hypothesized that a gain of function of *GLI1* would stimulate HH signaling, leading to an increase in bone formation. *PLXNB2* codes for a cell surface receptor that regulates different cellular processes, particularly in nervous system development.^(^ ^74^ ^)^ Recently, several evidences point to an important role in bone. Plexin B2 (PLXNB2) is a receptor for semaphorin 4D (SEMA4D), an important ligand for bone remodeling.^(^ ^75^ ^)^ Indeed, the Sema4d‐KO mouse featured high bone mass, increased resistance to fracture, greater bone formation rate, and normal osteoclastic activity.^(^ ^76^ ^)^ Moreover, Zhang and colleagues^(^ ^77^ ^)^ demonstrated that PLXNB2 promotes osteogenic differentiation through the activation of the RhoA signaling pathway.

In conclusion, we have identified two promising missense variants in *VAV3* and *ADGRE5* that could play a role in defining the high BMD in combination with other variants which might enhance their effects. Further work will be necessary to assess the pathological role of these two variants. If these findings are confirmed, *VAV3* and *ADGRE5* might turn out to be novel therapeutic targets for the treatment of osteoporosis.

## Conflict of Interest

Service on Advisory Board: DO: Kyowa Kirin; XN: Amgen, UCB. Honoraria or royalties for books or publications or for lectures (speaker fees) or participating in a speakers bureau: DO: Speaker fees in a couple of talks for Kyowa Kirin; XN: Speaker fees from Amgen, Lilly. Research grants, direct salary support or other financial support from commercial entities: DO: Research grant funded by Kyowa Kirin. The other authors declare no conflict of interest regarding the research and results presented in it.

## Data Accessibility Statement

All data and material will be available upon request.

## Ethics Approval and Consent to Participate

All procedures performed were in accordance with the 1964 Helsinki declaration and its later amendments or comparable ethical standards. Both the Bioethics Committee of Universitat de Barcelona and the Clinical Research Ethics Committee of Parc de Salud Mar have emitted a favorable bioethical statement regarding the present research. Written informed consents were obtained from the participants in both instances.

### Peer Review

The peer review history for this article is available at https://publons.com/publon/10.1002/jbm4.10602.

## Supporting information


**Supplementary Table S1.** WES control quality stats.
**Supplementary Table S2.** Variants shared by HBM I.2, II.5 and III.1 and absent in II.1., III.2, III.3.
**Supplementary Table S3.** Variants shared by HBM I.2 and II.5 and absent in II.1., III.2, III.3.
**Supplementary Table S4.** Variants shared by HBM II.5 and III.1 and absent in II.1, III.2, III.3.
**Supplementary Table S5.** Variants by HBM II.5 and absent in II.1., III.2, III.3.
**Supplementary Fig. S1.** Pipeline filtering scheme. HSF: Human splicing finder http://umd.be/; MAF: Minor allele frequency; CADD https://cadd.gs.washington.edu/; SIFT indels https://sift.bii.a-star.edu.sg/, PROVEAN indels http://provean.jcvi.org.
