# Supplementary material for: Genetic Analysis in a Familial Case With High Bone Mineral Density Suggests Additive Effects at Two Loci
Source: JBMR Plus. 2022 Feb 18;6(4):e10602. doi: 10.1002/jbm4.10602 (PMC9009133; doi:10.1002/jbm4.10602)
Supplement: Supplementary file 1 — Supplementary Table S1. WES control quality stats. Supplementary Table S2. Variants shared by HBM I.2, II.5 and III.1 and absent in II.1., III.2, III.3. Supplementary Table S3. Variants shared by HBM I.2 and II.5 and absent in II.1., III.2, III.3. Supplementary Table S4. Variants shared by HBM II.5 and III.1 and absent in II.1, III.2, III.3. Supplementary Table S5. Variants by HBM II.5 and absent in II.1., III.2, III.3. Supplementary Fig. S1. Pipeline filtering scheme. HSF: Human splicing finder http://umd.be/; MAF: Minor allele frequency; CADD https://cadd.gs.washington.edu/; SIFT indels https://sift.bii.a-star.edu.sg/, PROVEAN indels http://provean.jcvi.org. [file JBM4-6-e10602-s001.docx]

**Supplementary Table 1.** WES control quality stats

| Sample | PF_Mreads | Uniquely_Aligned | Total_Duplicates | ROI_Size | Raw_Mean  Coverage | Effective_Mean  Coverage |
| --- | --- | --- | --- | --- | --- | --- |
| I.1 | 79.22 | 84.94 | 2.93 | 44637207 | 179.90 | 153.17 |
| II.2 | 95.80 | 86.79 | 2.98 | 44637207 | 212.24 | 186.66 |
| II.5 | 96.47 | 85.55 | 3.25 | 44637207 | 226.34 | 191.30 |
| III.1 | 49.79 | 95.06 | 28.25 | 40999737 | 128.54 | 82.80 |
| III.2 | 58.14 | 95.44 | 16.09 | 40999737 | 226.50 | 137.25 |
| III.3 | 43.42 | 95.45 | 12.92 | 40999737 | 160.81 | 108.18 |

**Supplementary Table 2.** Variants shared by HBM I.2, II.5 and III.1 and absent in II.1., III.2, III.3

| Gene | Variant | rs number | BMD GWAS | OMIM  Disease | Pathogenicity prediction | | | | MAF  gnomAD |
| --- | --- | --- | --- | --- | --- | --- | --- | --- | --- |
|  |  |  |  |  | **CADD** | **PP** | **PV** | **SIFT** |  |
| *ARMC9* | p.R536C^1^ | rs201558091 | N | JBTS30 | 26.3 | D | D | D | 0.000007954 |
| *RPUSD1* | p.T129N | NA | N | N | 23.3 | P | N | T | NA |
| *TBL3* | p.R690Q | rs79412280 | Y | N | 23.2 | P | N | T | 0.003560 |
| *HSPA12B* | p.H401D | rs1207415930 | N | N | 21.9 | B | N | T | 0.000008174 |

List sorted by CADD score. ^1^ NM_001352754.2; BMD GWAS: Genes associated with BMD in GWAS; Y: Yes; N: No; DISEASE: Gene associated with human diseases in OMIM: N: No; JBTS30: Joubert syndrome 30; CADD: http://cadd.gs.washington.edu; PP: Polyphen-2 http://genetics.bwh.harvard.edu/pph2/ D: Probably damaging; B: Benign; P: Possibly damaging; PV: PROVEAN http://provean.jcvi.org/; D: Deleterious; N: Neutral; SIFT: https://sift.bii.a-star.edu.sg/; D: Deleterious; T: Tolerated; MAF: minor allele frequency from gnomAD V2.1.1.

**Supplementary Table 3.** Variants shared by HBM I.2 and II.5 and absent in II.1., III.2, III.3

| Gene | Variant | rs number | BMD GWAS | OMIM Disease | Pathogenicity prediction | | | | MAF  gnomAD |
| --- | --- | --- | --- | --- | --- | --- | --- | --- | --- |
|  |  |  |  |  | **CADD** | **PP** | **PV** | **SIFT** |  |
| *KCNA10* | p.R153C | rs41281370 | N | N | 27.7 | D | D | D | 0.0008664 |
| *AMOTL1* | p.Y605S^1^ | . | N | N | 26.5 | D | D | D | NA |
| *VAV3* | p.T124I | rs200980013 | N | N | 24.2 | B | N | T | 0.0001497 |
| *ASB16* | p.A218V | rs75036136 | Y | N | 24.1 | D | D | D | 0.001149 |
| *TEKT3* | p.R183W | rs201038012 | N | N | 23.4 | D | D | D | 0.00006741 |
| *TFAP2E* | p.G16E | rs200729597 | N | N | 23 | B | N | D | 0.0002375 |
| *CCDC178* | p.R472W | rs146565345 | N | NA | 22.8 | D | D | D | 0.001100 |
| *CDK5RAP3* | p.D271N^2^ | rs140552517 | Y | N | 22.6 | B | D | T | 0.002379 |
| *ST14* | p.A744V | rs142090589 | N | ARCI11 | 24.8 | P | N | T | 0.0006158 |
| *PRR15L* | p.G99R | rs151133495 | Y | N | 22.3 | B | N | D | 0.0001559 |
| *KBTBD7* | p.H269Y | rs148638355 | N | N | 21.9 | P | N | D | 0.001478 |

List sorted by CADD score. ^1^ NM_130847.3; ^2^ NM 176096.3; BMD GWAS: Genes associated with BMD in GWAS; Y: Yes; N: No; DISEASE: Gene associated with human diseases in OMIM: N: No, NA: Not available; ARCI11: Ichthyosis, congenital, autosomal recessive 11; CADD: http://cadd.gs.washington.edu; PP: Polyphen-2 <http://genetics.bwh.harvard.edu/pph2/>; D: Probably damaging; B: Benign; P: Possibly damaging; PV: PROVEAN <http://provean.jcvi.org/>; D: Deleterious; N: Neutral; SIFT: <https://sift.bii.a-star.edu.sg/>; D: Deleterious; T: Tolerated; MAF: minor allele frequency from gnomAD V2.1.1.

**Supplementary Table 4.** Variants shared by HBM II.5 and III.1 and absent in II.1, III.2, III.3

| Gene | Variant | rs number | BMD GWAS | OMIM  Disease | Pathogenicity prediction | | | | MAF  gnomAD |
| --- | --- | --- | --- | --- | --- | --- | --- | --- | --- |
|  |  |  |  |  | **CADD** | **PP** | **PV** | **SIFT** |  |
| *GLIPR1L2* | p.W163C | rs149571330 | N | N | 31 | D | D | D | 0.00009859 |
| *VPS13B* | p.A3691T^1^  p.A3716T^2^ | rs142476821 | Y | COH1 | 28.8 | D | D | D | 0.002538 |
| *WDR41* | p.G61D | rs389319 | N | N | 26 | D | D | D | 0.002270 |
| *DECR1* | p.D287G^3^ | rs148549954 | N | N | 24.9 | B | D | D | 0.001663 |
| *ADGRE5* | p.R794W^4^ | rs369617596 | N | N | 23.7 | D | D | D | 0.0001279 |
| *ERAP1* | p.V647I | rs111363347 | N | N | 23.7 | D | N | D | 0.002911 |
| *OR10H3* | p.Y121C | rs114904510 | N | NA | 23.7 | D | D | D | 0.001708 |
| *RAP1GAP* | p.T183A^5^ | rs142233496 | Y | N | 23.4 | B | N | T | 0.001668 |
| *GLI1* | p.R510W^6^ | rs149817893 | N | PA | 22.8 | B | N | D | 0.002683 |

List sorted by CADD score. ^1^ NM_152564.5; ^2^ NM_017890.5; ^3^ NM_001359.2; ^4^ NM_078481.4; ^5^ NM_002885.4; ^6^NM_005269.3; BMD GWAS: Genes associated with BMD in GWAS; Y: Yes; N: No; DISEASE: Gene associated with human diseases in OMIM: N: No, NA: Not available; COH1: Cohen syndrome; PA: Polydactyly; CADD: <http://cadd.gs.washington.edu>; PP: Polyphen-2 http://genetics.bwh.harvard.edu/pph2/; D: Probably damaging; B: Benign; PV: PROVEAN http://provean.jcvi.org/; D: Deleterious; N: Neutral; SIFT: <https://sift.bii.a-star.edu.sg/>; D: Deleterious; T: Tolerated; MAF: minor allele frequency from gnomAD V2.1.1.

**Supplementary Table 5.** Variants by HBM II.5 and absent in II.1., III.2, III.3

| Gene | Variant | rs number | BMD GWAS | OMIM  Disease | Pathogenicity prediction | | | | MAF  gnomAD |
| --- | --- | --- | --- | --- | --- | --- | --- | --- | --- |
|  |  |  |  |  | **CADD** | **PP** | **PV** | **SIFT** |  |
| *ZBTB21* | p.R926H^1^ | rs762954242 | N | N | 28 | D | N | D | 0.000004046 |
| *BPGM* | p.D150Y | rs374333584 | Y | ECYT8 | 25.7 | P | D | D | 0.00007163 |
| *HMCN1* | p.G1224R | rs768384278 | N | ARMD1 | 25.5 | D | D | D | 0.00003187 |
| *PLXNB2* | p.E1804K | rs149124212 | Y | N | 25.4 | D | N | D | 0.004006 |
| *NPDC1* | p.R11Q | NA | N | N | 25.2 | D | N | D | NA |
| *C1orf159* | p.F130V^2^ | rs201998414 | N | NA | 24.5 | D | D | D | 0.0001420 |
| *KLRG2* | p.P154L | rs141268710 | N | NA | 23.8 | D | N | D | 0.001391 |
| *USP36* | p.K306R | rs200324750 | Y | N | 23.1 | B | D | T | 0.0002404 |
| *IQCN* | p.R496L^3^ | rs138404210 | Y | NA | 23 | D | D | D | 0.002429 |
| *PIK3R2* | p.V54M | rs201370957 | Y | MPPH1 | 23 | D | N | D | 0.002038 |
| *HMCN1* | p.D4389G | rs1033280778 | N | ARMD1 | 22.4 | D | N | T | NA |
| *ATG9B* | p.G8W | rs191759925 | Y | N | 22.3 | D | NA | NA | 0.0007790 |
| *ZNF813* | p.K552N | rs186777604 | N | NA | 21.8 | D | D | D | 0.001136 |
| *FAM160B1* | p.F572fs | NA | N | N | NA | NA | NA | NA | NA |

List sorted by CADD score. ^1^ NM_001098402.2; ^2^ NM_017891.5; ^3^ NM_001145304.2;BMD GWAS: Genes associated with BMD in GWAS; Y: Yes; N: No; DISEASE: Gene associated with human diseases in OMIM: N: No, NA: Not available; ARMD1: Macular degeneration, age-related, 1; ECYT8: Erythrocytosis, familial, 8; MPPH1: Megalencephaly-polymicrogyria-polydactyly-hydrocephalus syndrome 1; CADD: <http://cadd.gs.washington.edu>; PP: Polyphen-2 http://genetics.bwh.harvard.edu/pph2/; D: Probably damaging; B: Benign; P: Possibly damaging; PV: PROVEAN <http://provean.jcvi.org/>; D: Deleterious; N: Neutral; SIFT: <https://sift.bii.a-star.edu.sg/>; D: Deleterious; T: Tolerated; MAF: minor allele frequency from gnomAD V2.1.1.


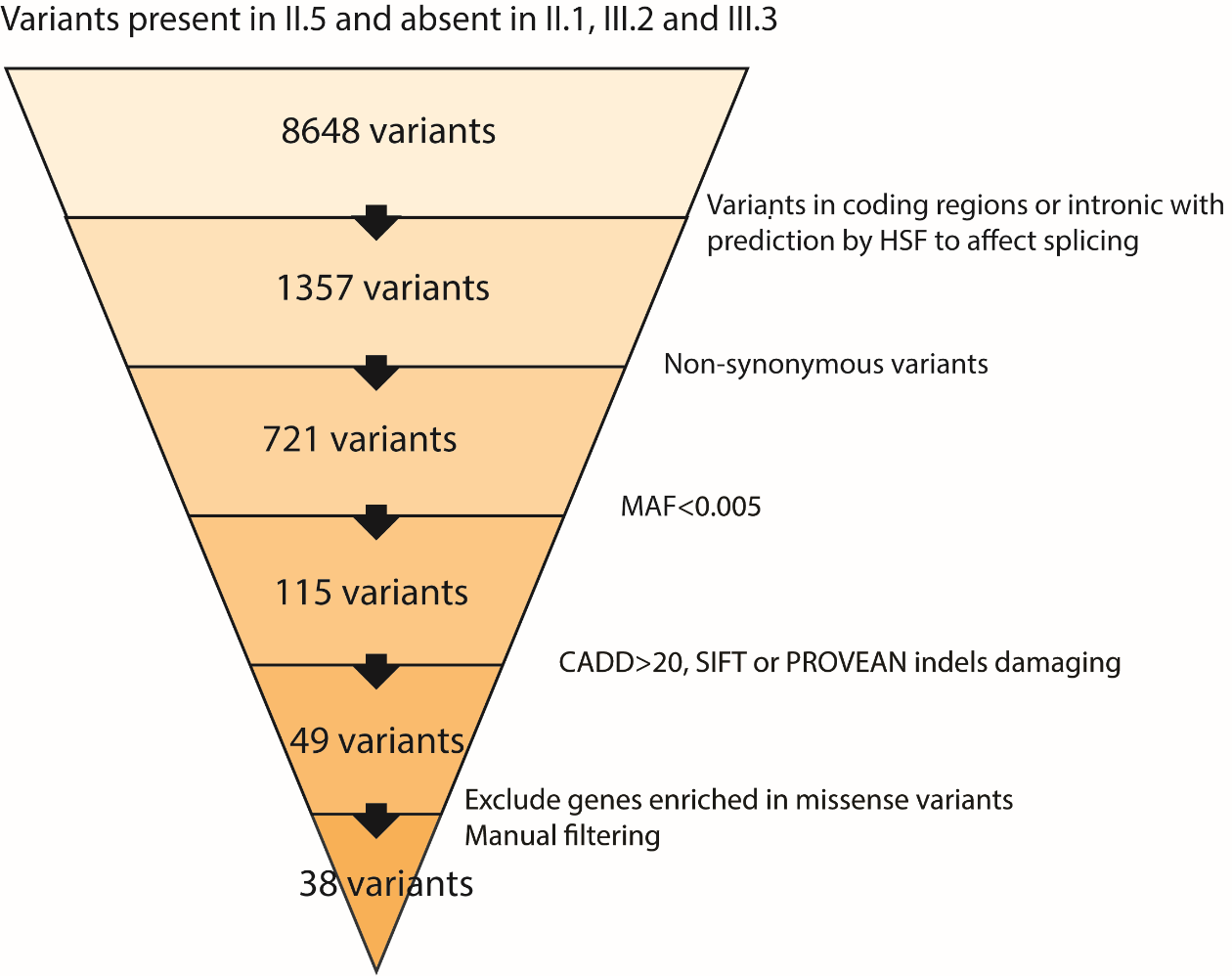


**Supplementary Figure 1.** Pipeline filtering scheme. HSF: Human splicing finder <http://umd.be/>; MAF: Minor allele frequency; CADD <https://cadd.gs.washington.edu/>; SIFT indels <https://sift.bii.a-star.edu.sg/>, PROVEAN indels http://provean.jcvi.org/
